# Visual Network Analysis Based on Stereo Vision and Feature Matching Algorithm

**DOI:** 10.1155/2022/2910531

**Published:** 2022-06-28

**Authors:** Lili Shi

**Affiliations:** Qingdao Huanghai College, Qingdao, Shandong 266000, China

## Abstract

Functions such as Internet browsing and online shopping have a great impact on people's lives. The footprints of people browsing various information and news on the web page are also increasing year by year. More and more people begin to pay attention to the visual communication in web design. Network information is widely loved by people because of its convenience, quickness, and simplicity. In order to study the visual problem in network information, this paper proposes to use a feature matching algorithm to study the visual information transmission in web design. Using stereo vision and feature matching algorithm, the target recognition function in the visual communication of web design is realized. The content of web design is defined from the perspective of visual beauty and overall harmony. By extracting the feature points in the data, the two-dimensional vision is transformed into a three-dimensional vision. It not only can accurately extract feature points from data but also can convert 2D vision to 3D vision. Finally, in order to optimize the feature matching speed of web design images, the epipolar constraint algorithm is used to optimize the feature matching function. The experimental results show that the content of this paper can truly show the virtual effect in web design and can intuitively upload and transmit simple and rich information content. This paper not only meets the aesthetic needs of the public for web design but also improves the problem of fuzzy information in the process of visual information transmission.

## 1. Introduction

Web design is the effect of information transmission through the combination of network technology and computer technology with visual elements [1]. When browsing websites and various web pages, people usually judge and evaluate the beauty and visual experience of web pages. If network information wants to become the main way of multimedia information transmission, it is necessary to optimize and update the field of Web Design [2]. Focus on the effect of visual communication and people's experience. Make visual communication and web design meet the needs of the masses. Therefore, we need to analyze various visual elements in website construction and web page design to determine the impact of visual effects on people's physiological needs [3]. Professional website designers also need to investigate design schemes with high public feedback and integrate visual elements into web browsing. By analyzing and evaluating the connection, location, and feedback of visual senses, researchers explored the human visual process. This paper mainly analyzes the internal structure of the eye in the process of human vision [4]. When we observe objects and browse the web, we only focus on the objects we are interested in. Focus on the focus first and then pay attention to the surrounding environmental factors [5]. Therefore, the browsing process is a dynamic sequential structure, and there is order and direction. We can judge the current psychological activities and interest characteristics of users according to the sense of direction of visual browsing [6].

With the gradual improvement of the quality of people's daily life, there are richer requirements in aesthetic needs. Nowadays, web design has shifted from functionality to aesthetics [7]. It takes network information as data and a network platform as a carrier to transmit all kinds of news and newsletters to people. This convenient and fast way has been highly praised by everyone. It is precisely because people's aesthetic needs are gradually increasing that people's visual feelings need to be considered in web design [8]. If we need to beautify and improve the visual function, we should first ensure the simplicity of information content in web page design and serve the form of visual communication. Second, the information content should be displayed in the visual process to ensure a natural and smooth browsing feeling [9]. At the same time, the quantity of network information is controlled, and the overall layout is planned uniformly. Finally, in web design, we need to ensure that people can focus on the information content, and cannot miss the key content because of the surrounding environment or useless information on the web page. In order to meet the above problems in the visual communication of web design, we use a feature matching algorithm for research and analysis [10].

This paper is mainly divided into three parts. The first part briefly analyzes the visual information transmission function of web design and explores the application status of the feature matching algorithm in various countries. The second part first analyzes various elements in web design and users' needs for visual elements. Adopt stereo vision to transform people's traditional perception of web design and develop from 2D design to 3D design. Second, the point target recognition function of the feature matching algorithm on visual information is studied. Finally, in order to further improve the visual information transmission effect, we use the epipolar constraint algorithm to optimize it. Explore the effect of web page design optimized by feature matching algorithm. The third part analyzes the results of the research on visual communication of web design based on feature matching optimization algorithm.

## 2. Related Work

At present, web designers have a poor understanding of the role and importance of visual communication information and have not formed a deep understanding [11]. With the continuous development of information technology, they found that the use of web visual elements is not much in their application and research. Therefore, in the face of the slow development of visual information communication, we analyze the problems of web design at this stage. First, the visual expression function of web design is relatively single, and the amount of data of news information is large, which makes it difficult to typeset and classify. The accumulation of website information is a difficult problem for most designers. This not only limits the network communication function but also causes great trouble to visitors. The visual expression of web pages is also relatively single, which cannot attract users' interest in form. Second, although web design needs to accurately express information, most websites are not beautiful enough. The aesthetics of web pages is the core content of visual information transmission. We need to improve the typesetting efficiency of web information and reasonably match the color structure [12]. Finally, in the problem analysis, we found that the interaction between websites and web pages and users is too poor to help users obtain information quickly. People are easily affected by visual elements, resulting in distraction. Facing the above problems, we explore the function and function of the feature matching algorithm.

Information technology in the United States has developed rapidly, and they have made many achievements in the research of image feature detection [13]. The process of feature point detection and matching in computer vision tasks is a complex structure. Compared with the traditional image system, the image extraction system using a feature matching algorithm can label the scene information and improve the accuracy of feature detection.

The research cost invested by Germany in the field of UAV is large [14]. In order to meet the concept of low-altitude photography, they use UAV images to complete the photography link. However, in practical application, there are some problems with the image data taken by UAV, such as small pixels, more interfering objects, small photographing range, and so on. Therefore, they use a feature matching algorithm to optimize UAV photography. It improves the situation of image blur and many interference factors.

China's concept of green environmental protection is an important link to national sustainable development [15]. In the research of forestry information construction, there are high requirements for the accuracy of data resources and feedback efficiency. In the actual data acquisition, most of the information is easily disturbed by miscellaneous data. Therefore, the traditional forestry information construction still depends on the manual mode. With the development of information technology, researchers use feature matching algorithms to improve the time-consuming links in forestry work. It not only improves the accuracy of data information but also provides optimization and guarantees the processing efficiency of construction work. According to the above research on the development status of the feature matching algorithm in various countries, we have applied this algorithm to the analysis of visual information communication in web design and achieved good results.

## 3. Methodology

### 3.1. Research on Stereo Vision Based on Feature Matching Algorithm to Realize Point Target Recognition in Web Page Design

Information communication in web design is an important part of media communication. With the rapid development of network technology, the forms of visual communication have also changed. Technology is the basis of realizing the visual effect of web pages. Designers should take the initiative to master various existing network technology laws, pay attention to the close combination of technology and art, make full use of the advantages of technology, realize artistic imagination, and achieve ideal visual effects. Excellent layout design expresses harmony and beauty through the spatial combination of words and graphics. It can optimize reading, accurately transmit information, and make the website have an affinity. In web design, different colors should be combined and matched according to the principles of harmony, balance, and emphasis. In the network age, website and web page design need to meet the premise of functionality and ensure vision and aesthetics. At present, the focus of web design is still biased towards the literal mode, and the designer's technology and thinking are also limited. The visual expression of web pages is relatively single, not beautiful and advanced enough. In order to improve the conversion from the original two-dimensional image target to a three-dimensional image, we use the point target recognition technology of stereo vision to optimize the web design. First, the 3D information in the data is obtained; the 3D visual model is established; and the boundary of the data points is judged and analyzed. The acquisition of point data is mainly recorded by laser scanning. In order to better store and match data, we use a ring network for the construction of three-dimensional data points. First, the target position of the data is defined as the origin of the coordinate system, and the vertical parameters are injected into the coordinate system. Through the above operations, we can obtain the optimal position distance of visual information points. In order to meet the coverage and accuracy of three-dimensional direction, we express the optimal distance as

(1) $$\begin{array}{c} D_{\text{best}}=\min\left[\sqrt{{\left(x_{i}-x_{o}\right)}^{2}+{\left(y_{i}-y_{o}\right)}^{2}+{\left(y_{i}-x_{o}\right)}^{2}}\right], \end{array}$$

where *i* and *o* represent the target point coordinates and the simulation coordinate system, respectively. The minimum distance in all positions can be obtained by calculating the three directions corresponding to the feature points. Because the position of 3D target points will involve attitude problems, we need to model the target point data to test the specific position of the target in the coordinate system. The center relationship of the datum line can be expressed as follows:

(2) $$\begin{array}{c} X_{i}=\left(a+D_{\text{best}}\right)\cos\left(\alpha_{i}\right)\cos\left(\beta_{i}\right),\\ Y_{i}=\left(b+D_{\text{best}}\right)\sin\left(\beta_{i}\right),\\ Z_{i}=\left(c+D_{\text{best}}\right)\sin\left(\alpha_{i}\right)\cos\left(\beta_{i}\right), \end{array}$$

where *X*_*i*_, *Y*_*i*_, and *Z*_*i*_ is the center position parameter of the datum line, which can calculate the correction range of the optimal distance. *α*_*i*_ and *β*_*i*_ represent the offset value of the corresponding target point. Through the test of the offset value, we can judge whether there is a difference in the angle.

**Figure 1:**
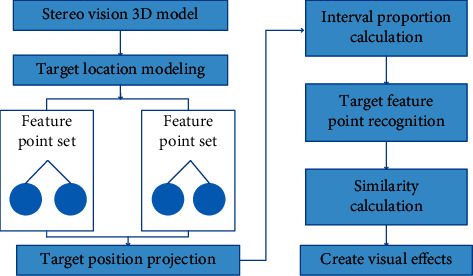
Flow chart of stereo vision target recognition.

The essential concept of visual stereo is to reorganize the three-dimensional coefficients of the target to be measured by analyzing the three-dimensional structure of the feature point target. In the actual recognition process, because the target has an external structure, the model of the sample data can be used as a reference to judge the position of the target point. In the research on the influencing factors of the stereo vision effect, the most obvious influencing parameter is the angle of the actual position. The distributed recognition effect can be obtained by projecting the angles of different samples, which can improve the success rate of sample feature target recognition. The specific operation flow is shown in Figure 1.

Feature points are those points that are analyzed by the algorithm and contain rich local information. The so-called “scale invariance” of feature points refers to their unified properties that can be recognized in different pictures. As can be seen from Figure 1, first, the position of the detection target is modeled to form different types of feature point sets. The target is projected according to the feature point set. Second, in the projection interval of each feature point, the appropriate proportion is selected for calculation. The final data obtained by calculation is the selected proportional value. After selecting the target sample test area, traverse and filter. Finally, target recognition is carried out, and the number of features is obtained from the target set of multiple structures. The false target is eliminated by similarity calculation to obtain the final stereo vision effect.

In practice, the target interval to be selected can be represented by any parameter. When the laser scanning is projected to the target, the distance parameter between samples can be obtained. Because of stereo vision, 3D style changes from 2D data. Therefore, if there is a given target at the corresponding projection angle, the stacking data along the fuzzy position of the target can obtain the specific position. If the projection distance is too large, the value calculated by traversal is obtained according to a certain proportion. The data of target points is in three-dimensional mode. Before completing the construction of stereo vision, the angle of two-dimensional to three-dimensional mapping needs to be selected. It is known that the laser direction is a fixed parameter and the direction is the real distance of the projection. Therefore, the laser direction is selected as the projection direction when extracting the feature of the target point set. In order to optimize the two-dimensional style of web design, the data of sample parameters in feature points are complex. These eigenvalues can automatically form any matching matrix. We calculate the eigenvalues of the matrix to obtain the actual matching relationship between targets. To determine whether the target conforms to the three-dimensional type, the matrix expression is

(3) $$\begin{array}{c} A={\left(r-1\right)}^{-1}C^{T}C=\left(\begin{array}{c} \mathrm{cov}\left(x,x\right)\mathrm{cov}\left(x,y\right)\\ \mathrm{cov}\left(y,x\right)\mathrm{cov}\left(y,y\right) \end{array}\right),\\ A={\left(r-1\right)}^{-1}C^{V}C=\left(\begin{array}{c} \mathrm{cov}\left(x,y\right)\mathrm{cov}\left(x,z\right)\\ \mathrm{cov}\left(y,y\right)\mathrm{cov}\left(y,z\right) \end{array}\right),\\ A=\left(2r-1\right)C^{V}C^{T}=\left(\begin{array}{c} \mathrm{cov}\left(x,y\right)\mathrm{cov}\left(x,z\right)\\ \mathrm{cov}\left(y,y\right)\mathrm{cov}\left(y,z\right)\\ \mathrm{cov}\left(z,y\right)\mathrm{cov}\left(z,z\right) \end{array}\right). \end{array}$$

**Figure 2:**
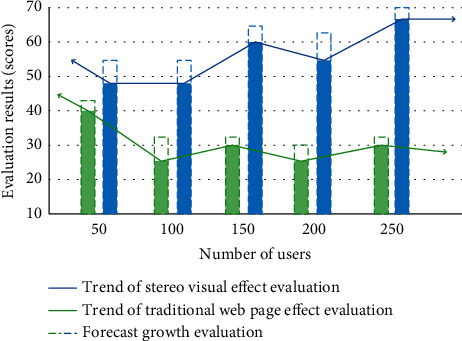
Comparison of evaluation before and after optimization.

In (3), the matrix has a covariance form; this structure is symmetrical; and the calculation results can be obtained by eigenvector decomposition. For different characteristic functions, the actual positions of test points are similar. In order to distinguish effectively, we use a similar function calculation:

(4) $$\begin{array}{c} r\left(\text{fp},\text{fQ}\right)=\frac{n\sum \text{fpfQ}-\sum \text{fp}\sum \text{fQ}}{\sqrt{n\sum f^{2}p-{\left(\sum \text{fp}\right)}^{2}}},\\ r\left(\text{fp},\text{fQ}\right)=\sqrt{n\sum f^{2}Q-{\left(\sum \text{fQ}\right)}^{2}}, \end{array}$$

where *p* and *Q* represent two test target points, and fp and fQ are the eigenvalue set range corresponding to the two targets. *n* represents the number of calculated eigenvectors. In order to improve the accuracy of target recognition, we bring stereo vision into the feature matching algorithm of boundary value calculation and filter the position according to the value range in the feature matching process. Only the data that conforms to the defined range of the target can be matched, which greatly improves the information accuracy of the visual effect. The overall beauty of the web page design optimized by stereo vision has been improved. We investigated this and formed a comparative evaluation, as shown in Figure 2.

As can be seen from Figure 2, we selected loyal customers who have used a website for a long time as variable parameters and the evaluation results as control variables. The stereo vision effect optimized by the feature matching algorithm has achieved good evaluation feedback in web page design. Compared with the visual communication effect of traditional web design, the research content of this paper is effective.

### 3.2. Research on Visual Information Communication of Web Design Based on Feature Matching Algorithm Optimization

Visual communication design plays a very important role in improving the competitiveness of Chinese enterprises. The development speed is very rapid in the intersection and blending with many disciplines. Due to the differences in solving problems and different ways of solving problems, different knowledge structures are required, so new majors are bound to appear and develop. Once the design is simplified by computers and other modern equipment, most of the designer's time will not be spent on the completion work. The formation of ideas plays an important role. The design and function of web pages can help people get information, and the importance of visual communication cannot be ignored. Visual information transmission should take the transmission speed of information as the primary research content and improve the accuracy of the information in practical optimization. Finally, combined with the aesthetic trend of the masses, the web page design is optimized and improved. In the environment of the big data era, web pages will produce news information with a huge amount of data every day. Concise typesetting and layout are very important links in web design. In order to ensure that users can easily obtain the required content, we consider simplifying the processing of the information. However, in the process of information simplification, it is easy to reduce the accuracy. In this regard, our research needs to ensure the accuracy of information and meet the visual effect of web design. In order to improve the matching speed and accuracy of information content, we optimize the feature matching algorithm. A matching algorithm RANSAC algorithm based on epipolar constraint is proposed, which processes the feature point data in the information and processes the information normally. The original feature matching uses the stable data feature points in the information to compress the amount of information processing to achieve the effect of search and matching. This search mode has less computation and is easier to complete the specified task. However, in the big data environment, such methods can no longer meet the current needs of people. Therefore, we use the epipolar constraint matching algorithm to optimize and summarize the information feature points into the same epipolar, which greatly reduces the scope of matching and search. The feature point basis vector matches the feature points to explore the relationship between them. The calculation results can directly affect the accuracy of the matching information. We normalize the information data and convert the original information to reduce the impact of interference data. RANSAC parallel algorithm is used to improve the speed of calculation. First, change the location of the matching information and standardize the location. Assuming that the parameters of any feature point are a function value, the processed information points are

(5) $$\begin{array}{c} M_{l}=T_{l}m_{l},M_{r}=T_{r}m_{r},\\ M_{l}=T_{l}m_{l},M_{r}=T_{r}m_{r}, \end{array}$$

in which

(6) $$\begin{array}{c} T_{l}=\left[\begin{array}{c} k0-k_{m}u_{0}\\ 0k-k_{m}v_{0}\\ 0\ldots .0\ldots ..1 \end{array}\right],\\ u_{0}=\frac{1}{N}\sum_{i=1}^{N}u_{i},\\ v_{0}=\frac{1}{N}\sum_{i=1}^{N}v_{i},\\ \text{km}=\frac{\sqrt{2}}{\sqrt{\left(1/N\right)\sum_{i=1}^{N}\left[{\left(u_{i}-u_{0}\right)}^{2}+{\left(v_{i}-v_{0}\right)}^{2}\right]}}. \end{array}$$

**Figure 3:**
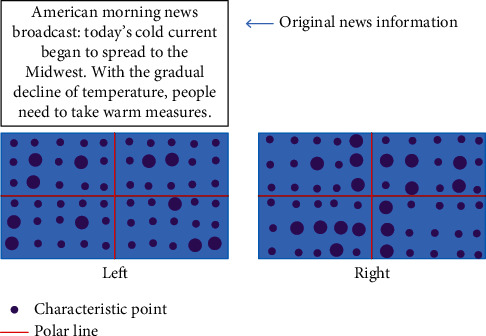
Position relationship between sample feature points and epipolar lines.

In order to improve the efficiency of information computing, we use parallel computing to obtain matrix results. Record the maximum number of values in each group and the corresponding basic matrix and make statistics by sampling count:

(7) $$\begin{array}{c} m=\frac{\log\left(1-p\right)}{\log\left[1-{\left(1-\lambda \right)}^{8}\right]}, \end{array}$$

where *p* represents the probability that at least one of *m* sample points is accurate information and *λ* represents the error rate of matching. The distance between the data information matching points is calculated according to the set characteristics of the epipolar line, and the calculation results are used to express the deviation variable. The corresponding formula is

(8) $$\begin{array}{c} d_{i}=d_{l}+d_{r}=d\left(m_{l1},F^{T}m_{r2}\right)+d\left(m_{l2},{\text{Fm}}_{r1}\right), \end{array}$$

where *d*_*i*_ represents the distance from the information point to the polar line. We select a piece of information data in the sample web page as the test point. The positional relationship between sample feature points and epipolar lines is shown in Figure 3.

It can be seen from Figure 3 that the data in the left figure are scattered greatly before eigenvalue matching optimization, while the data after feature point matching optimization are related to the epipolar position. It can be seen that each calculation process of the RANSAC algorithm model is carried out independently. Selecting a large number of initial data of information samples can also improve the calculation speed of the algorithm.

## 4. Result Analysis and Discussion

### 4.1. Analysis of Research Results of Point Target Recognition Technology Based on Feature Matching Algorithm

**Figure 4:**
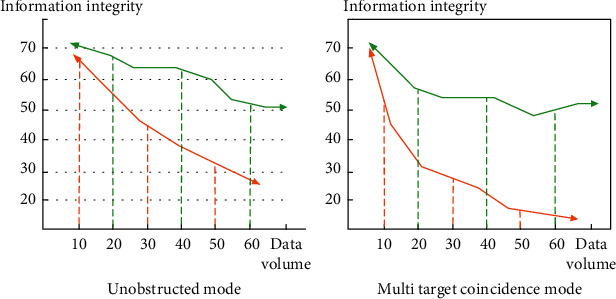
Comparison of integrity between stereo visual information expression and traditional visual expression.

Whether websites and web pages can convey effective information to users is affected by many factors. Among them, the visual communication effect is a very important link. The visual effect of the web page can bring users the impression that meets their aesthetic needs. It is the first environment for people to contact information. Information goes deep into the brain through visual communication functions, and people analyze and interpret information. Therefore, the concept and thinking of web design must comply with the visual information communication effect and visual communication process. The visual information transmission process is controlled by people's eye characteristics. Physiological conditions and eye structure are complex environments. We understand and absorb information through visual target capture. In web design, designers should pay attention to the visual effect of web pages. The traditional two-dimensional space effect can no longer meet the needs of modern society. In order to improve the visual information transmission effect of web pages, this paper uses a feature matching algorithm to upgrade the two-dimensional effect. The design method in this paper has a stronger sense of space in aesthetics and can bring more intuitive feelings to readers. In order to make a more accurate model, research has improved the visual information transmission effect of web pages. In the experimental process, laser scanning is used to process the point target data in the area. The test object is aimed at the image information inside the web page. Detect and recognize different images and types. First, the target point data is obtained by laser scanning. Then, the filtering operation is performed to obtain the image information marked by the feature points. Because the sample data to be tested is filtered for a long time, the sample points in line with visual aesthetics will be retained. Remove excess astigmatism, background, and other interference data and reanalyze the test image from various angles. Finally, the marked target is consistent. In order to test the recognition effect of different image information in web design. We control the experimental conditions and set two modes: multitarget coincidence and no occlusion. Explore the integrity of stereo vision effect on image information expression, as shown in Figure 4.

It can be seen from Figure 4 that the stereo vision studied by using the feature matching algorithm has the highest effect on the integrity of image information representation. The traditional visual information transmission function cannot effectively obtain and recognize the information in the face of multitarget coincidence. Therefore, the three-dimensional effect of the feature matching algorithm in stereo vision can restore image information in a complex environment, which contributes to the beauty and accuracy of web design.

### 4.2. Analysis of Research Results of Visual Information Communication in Web Design Based on Feature Matching Algorithm Optimization

**Figure 5:**
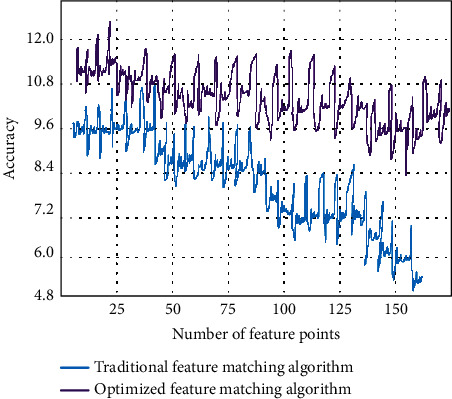
Comparison of information accuracy before and after feature matching algorithm optimization.

In order to improve the accuracy and efficiency of information acquisition in web design, the function of visual information transmission should also be optimized. Designers need to follow the visual and sensory characteristics of the masses, start from the information and data typesetting, and realize the aesthetic needs and interactive functions of the masses. In the face of more big data information, it is necessary to simplify the information data. Prevent complex environment and messy information from affecting users' attention. In the visual information transmission of web design, in order to improve the information accuracy and matching effect in the big data environment, we optimize the feature matching algorithm. RANSAC algorithm based on epipolar constraint is proposed to control the distance of feature points. In order to verify that the experimental algorithm improves the accuracy of information acquisition, we use the internal information of a web page in big data as the experimental data. Take the number of feature points of the target to be measured as the variation and explore the information accuracy before and after the optimization of the feature matching algorithm, as shown in Figure 5.

As can be seen from Figure 5, the experimental results of the two algorithms are very different. Due to the huge amount of data information, the traditional feature matching algorithm suffers from data loss when identifying information targets. This leads to a decline in the internal accuracy of information as the amount of information increases. The optimized feature matching algorithm not only can ensure the accuracy of information data but also can meet the visual aesthetics of the masses. Finally, this paper puts forward some strategies for visual information communication in web design. First, designers should pay attention to the main content and aesthetic needs of information on web pages. While ensuring users' perception of visual senses, highlight the key content of information. Highlight their own advantages and characteristics in website design and web page layout and bring a convenient and fast user experience to users. Second, the rational use of multimedia information technology not only can add picture elements to web design but also can add dynamic elements such as video and audio. This multimode design is more in line with people's visual experience than traditional text information. Finally, the interaction effect with users is added to web design. Let users change from passive acceptance of information to active acquisition.

## 5. Conclusion

Web browsing is the main way to obtain information in the era of big data. As a form of visual communication, web design can combine text, pictures, audio, and other elements and become an interactive tool to meet multidimensional feelings. Traditional web design can only meet the expression of information. With people's longing for a better life, this form of web design does not meet the needs of contemporary users. Based on the above situation, this paper uses the feature matching optimization algorithm to study the visual information transmission mode of web design. This paper analyzes and explores the situation of single web page arrangement, poor information transmission effect, and poor interactivity. First, the three-dimensional visual effect is used to obtain the target information on the web page. The main contents of the information are marked, and the matching function is optimized by a feature matching algorithm. Second, focus on the problem of recognition accuracy, highlight the target information from a variety of web pages, and realize the simple and fast function of visual communication. Finally, we find that the traditional feature recognition algorithm is easy to cause information loss when matching information in the big data environment. In this paper, the RANSAC algorithm is used to optimize feature matching and apply it to the visual information communication function. The experimental results show that the feature matching algorithm can accurately obtain the web page information and replan the stereo vision. Compared with the traditional two-dimensional mode, it has been greatly improved in design aesthetics. The optimized feature matching algorithm can also ensure the accuracy of the information in the case of too much web page data and improve users' experience of web pages. However, the research still has some limitations. This paper does not discuss the visual performance of the feature recognition algorithm in different web page features. Therefore, there is a problem of weak applicability. This needs further elaboration and analysis in future discussions.

## Data Availability

The data used to support the findings of this study are available from the author upon request.

## References

[1] Zhang Q. (2021). Research on text graphics in visual communication design. *Journal of Hubei open vocational college*.

[2] Wu Na (2021). Color matching of visual communication in web design. *Popular colors*.

[3] Zhang Z. (2021). Research on the application of visual communication in web design. *Art education research*.

[4] Cao Z. (2021). Expression and application of visual elements in web design. *China newspaper*.

[5] Zhao J. (2021). Application of information design in web visual communication. *Modern industrial economy and informatization*.

[6] Hui Li (2021). Research on text application based on visual communication design. *Journal of Jingdezhen University*.

[7] Sun M. (2021). Research on the application of visual elements in Web Design in the new media era. *Editing*.

[8] Zhang F., Li M. (2021). Plane visual communication design of user experience effect based on computer simulation. *Journal of Physics: Conference Series*.

[9] Guo H., Xuan L., Hao C., Kateb F., Alzyoud A. A. Y. (2021). Research on the mathematical model construction of art and ideology in visual communication design teaching. *Applied Mathematics and Nonlinear Sciences*.

[10] Qin H., Xiong J., Xu M. (2020). Research on the unity of visual communication and information transmission in web design. *Tomorrow fashion*.

[11] Sun H., Zheng T. (2020). Expression form of web design in visual communication design. *Western leather*.

[12] Zhang Z., Zhang Z., Zhu B. (2021). A fast algorithm for feature matching and target location based on Shi Tomasi and improved LBP. *Journal of Jilin University (Science Edition)*.

[13] Li Y., Zheng W., Liu X., Mou Y., Yin L., Yang B. (2021). Research and improvement of feature detection algorithm based on FAST. *Rendiconti Lincei-Scienze Fisiche e Naturali*.

[14] Ye K., he Y., Chi J., Zhu X., Yang F., Rui X. (2021). Research on visual SLAM algorithm based on optimal feature point extraction. *Industrial control computer*.

[15] Ren G., Liu P., He Z. (2021). Improved matching algorithm based on lidar feature extraction. *Journal of Shaanxi University of Science & Technology*.

